# Ginkgolide B Alleviates Learning and Memory Impairment in Rats With Vascular Dementia by Reducing Neuroinflammation *via* Regulating NF-κB Pathway

**DOI:** 10.3389/fphar.2021.676392

**Published:** 2021-06-17

**Authors:** Lijuan Huang, Yijie Shi, Liang Zhao

**Affiliations:** School of Pharmacy, Jinzhou Medical University, Jinzhou, China

**Keywords:** ginkgobalide B, vascular dementia, TLR4, NF-κB, inflammatory

## Abstract

Ginkgobalide B (GB) as the main active ingredient of traditional Chinese medicine Ginkgo biloba extract is reported to reduce neuroinflammation, protect neurons and promote cognitive learning ability. To explore that GB can reduce neuroinflammation through regulating nuclear factor-kappaB (NF-κB) signaling pathway and overcome cognitive dysfunction in rats with vascular dementia (VD), we aim at investigating the potential effect of GB on enhancing cognitive function in rats with VD. It was found that GB improved survival of oxygen-glucose deprivation (OGD) treated SH-SY5Y cells by attenuating inflammatory response via Toll-like Receptor 4 (TLR4)/NF-κB pathway. When rats were treated with bilateral common carotid artery occlusion (BCCAO) for 24 h, saline and GB were administered in Sprague-Dawley (SD) rats via a single intraperitoneal injection for consecutive 14 days. The behavioral changes of VD like rats treated with GB were observed through open field test, Morris water maze (MWM) and Y-maze electric maze. Nissl staining and immunofluorescence were used to observe changes of neurons in the hippocampus of rats. Western blot analysis was performed by detecting NF-κB pathway related inflammatory factors. The results found that GB can significantly improve the learning and memory ability of VD rats by reducing TLR4/NF-κB mediated neuroinflammation. In conclusion, GB seemed to be a potential drug for amelioration of learning and memory impairment in rats with VD.

## Introduction

Vascular dementia (VD) as a neurodegenerative disease is caused by the decrease or blockage of blood flow in the brain and leads to impaired cognitive function of brain tissue (Iadecola, 2013; Kalaria, 2018). Epidemiological studies have shown that VD has become the second common type of dementia after Alzheimer disease (AD) (O'Brien and Thomas, 2015). It is reported that repeated brain ischemia reperfusion and long-term chronic hypoperfusion are the main causes of VD (Smith, 2017). 10% of patients develop dementia shortly after stroke, and more than one-third of patients develop dementia after repeated stroke (Pendlebury and Rothwell, 2009). Nowadays, there is no effective medicine to treat VD. It has been reported that vanillic acid reduces memory impairment and hippocampal inflammation during cerebral hypoperfusion and reperfusion in rats (Khoshnam et al., 2018). Hwangryunhaedok-tang prevents neuronal damage and relieves vascular dementia in bilateral common carotid artery occlusion (BCCAO) model by improving cholinergic dysfunction and inhibiting neuroinflammatory response (Sohn et al., 2019). Therefore, it is urgent to better understand pathobiology of VD and explore effective neuroprotective agents for treating VD.

The pathogenesis of VD involves enhanced neuroinflammation by triggering oxidative stress, secreting more pro-inflammatory cytokines and inducing nuclear factor-kappaB (NF-κB) activation (Sharma and Sighn, 2012; Sun et al., 2019). NF-κB as a transcription factor has been activated and partly involved in neuroinflammation in VD. The inhibition of NF-κB pathway contributes to cognitive improvement in mouse or rats model of VD (Saggu et al., 2016; Wang et al., 2020). Studies have confirmed that when VD occurs, Toll-like receptor 4 (TLR4) as a transmembrane protein I can activate the expression of downstream nuclear transcription factor NF-κB. The domain of TLR4 in cytoplasm binds to the carboxyl end of MyD88, causing MyD88 to recruit a large number of downstream serine protein kinase Interleukin-1 Receptor-Associated Kinase (IRAK), which leads to the phosphorylation of IRAK. After dissociation from MyD88, phosphorylated IRAK binds to tumor necrosis factor receptor-associated factor 6 (TRAF6) and activates inflammatory NF-κB pathways (He et al., 2016), thus resulting in the release of a large number of inflammatory factors such as Tumor Necrosis Factor alpha (TNF-α) and Interleukin 6 (IL-6). These inflammatory mediators can further trigger the inflammatory response by increasing the phosphorylation of IkBa (I-kappa-B-alpha), and further activating phosphorylation of NF-κB p65. Therefore, activated NF-κB p65 can further promote the increase of protein expression levels of multiple pro-inflammatory factors such as TNF-α and IL-6. It forms a positive feedback loop, which enlarges the inflammatory response and aggravates the neuronal inflammatory response and neuron injury.

**FIGURE 1:**
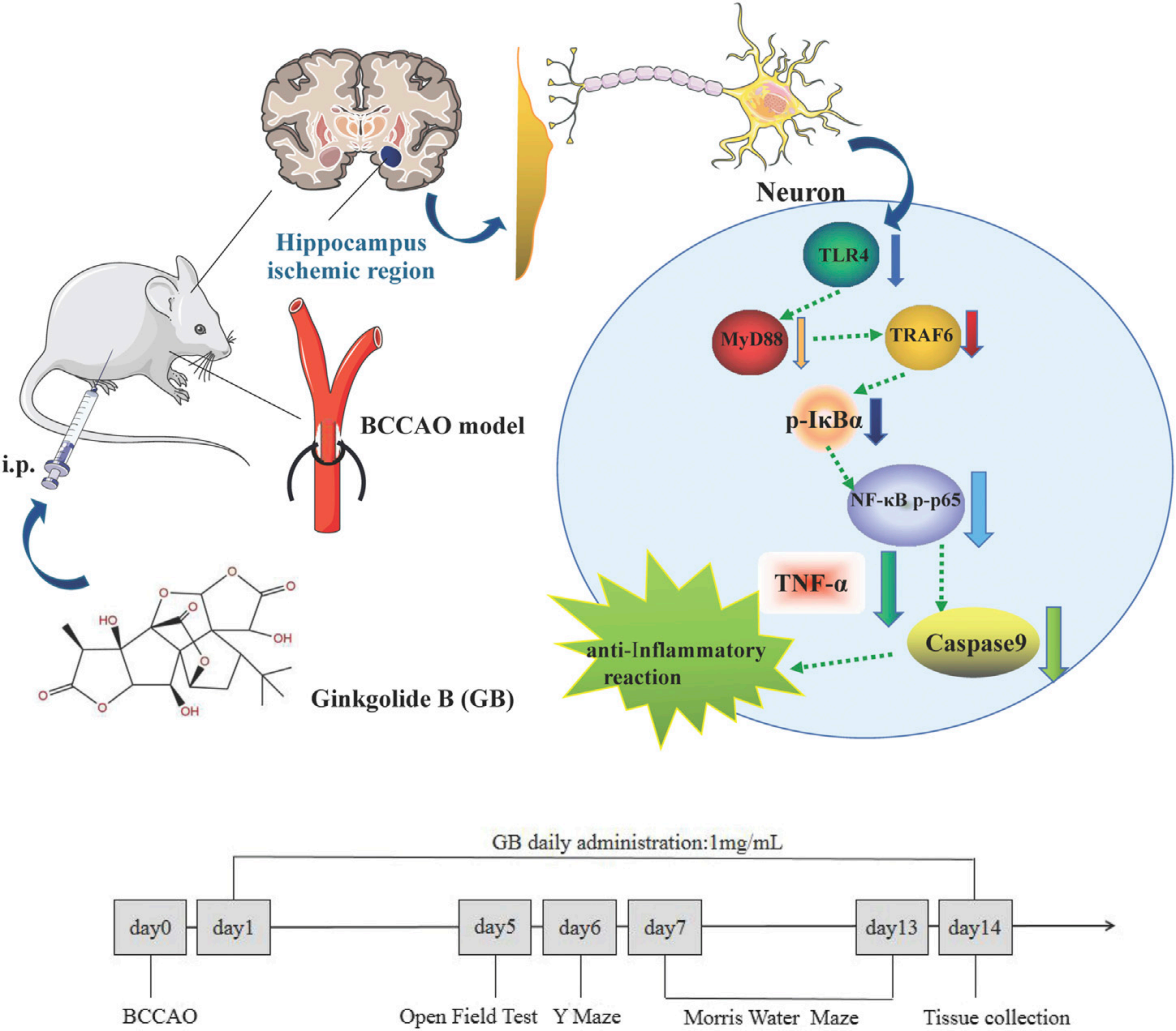
Graphical Abstract: Ginkgolide B alleviates learning and memory impairment in rats with vascular dementia by reducing TLR4/NF-κB mediated neuroinflammation.

Ginkgobalide B (GB) as the main active ingredient of Ginkgo biloba extract is recognized as a natural neuroprotective agent by reducing neuroinflammation, increasing brain blood flow and promoting learning and memory ability in several models of neurological diseases (Li et al., 2018; Rapp et al., 2018). Studies have shown that GB as platelet activating factor receptor (PAF-R) antagonist significantly deactivates the proinflammatory molecule NF-κB and reduces neuropsychotoxicity via a NF-κB dependent mechanism (Tran et al., 2018). Therefore, this study aims to explore whether GB can reduce neuroinflammation through regulating NF-κB signaling pathway and overcome cognitive dysfunction in rats with VD and graphical abstract was shown in Figure 1.

## Material and Animals

GB was purchased from Chengdu Must Bio-Technology Co., Ltd. Lipopolysaccharides (LPS) was purchased from Beijing Solarbio Science & Technology Co., Ltd. All other chemicals were of reagent level and used as received. The antibodies such as NF-κB p65, NF-κB p-p65, IκBα, p-IκBα, TLR4, MyD88, TRAF6, TNF-α and IL-6 (1:1000) were obtained from WanLeiBio (Shenyang, China) and the secondary antibody goat anti-rabbit IgG/HRP was obtained from EarthOx Life Sciences (Millbrae, CA, United States). Anti-NeuN (bs-10394R) was purchased from BEIJING BIOSYNTHESIS BIOTECHNOLOGY CO., LTD.

Male Sprague-Dawley (SD) rats (280–320 g) were provided by Jinzhou Medical University. The experimental protocol was performed with the approval of the Institutional Animal Care and Use Committee of Jinzhou Medical University (Protocol No. 2019008) and followed the National Guidelines for Animal Protection. Human neuroblastoma cells, SH-SY5Y cells were obtained from Chinese Academy of Sciences (Shanghai, People’s Republic of China) and cultured in Dulbecco’s Modified Eagle Medium: Nutrient Mixture F-12 (DMEM/F-12) supplemented with 10% fetal bovine serum (FBS) and 1% P/S.

### MTT and TUNEL Assay

SH-SY5Y cells were seeded in 96-well plates and subjected to 1 h of oxygen-glucose deprivation and 24 h of reoxygenation (OGD) according to the previous report (Meng et al., 2019). Cells were divided into five groups as follows: 1) Normal group: normal SH-SY5Y cells were treated with PBS. 2) OGD group: SH-SY5Y cells were exposed to OGD. 3) GB group: OGD exposed SH-SY5Y cells were treated with 20 μg/ml of GB. 4) LPS group: normal SH-SY5Y cells were treated with 100 ng/ml LPS. 5) LPS + GB group: normal SH-SY5Y cells were treated with 100 ng/ml LPS and 20 μg/ml of GB. After co-incubating with cells for 24 h, the medium was subsequently discarded and replaced with 200 μL of serum-free medium containing MTT (0.2 mg/ml; Sigma-Aldrich) and incubated for 6 h at 37°C at 5% CO_2_ and 95% O_2_. Then, the supernatant was aspirated, 150 μL of dimethyl sulfoxide was added to each well, and the absorbance was measured at 490 nm.

In order to evaluate GB mediated survival restoration of OGD treated SH-SY5Y cells, one step TUNEL apoptosis assay kit was used to detect the apoptosis level of cells according to the protocol. The DMI4000B fluorescence microscope used in the experiment came from Leica company (Germany).

## Induction of Bilateral Common Carotid Artery Occlusion Model *in vivo* and Drug Administration

The VD model was prepared with BCCAO (Kaundal et al., 2018). The rats were fasted for 8–12 h before surgery, freely drinking water, and intraperitoneally anesthetized with 10% chloral hydrate at 0.3 ml/100 g body weight. After anesthesia, rats were placed supine and fixed on the operating table. After shaving the neck and disinfecting with 75% alcohol, a median neck incision was performed. The common carotid artery was separated with surgical tweezers and occluded by inserting a 6–0 nylon monofilament suture followed by tying knots to prevent suture from falling off. The contralateral common carotid artery was treated with the same method. Finally, after the incision was sutured with no obvious abnormalities, rats returned to the cage for routine feeding. In the sham operation group, only bilateral common carotid arteries were separated without occlusion. According to the previous report (Zhang et al., 2020a), we selected 14 days duration of administration. The rats were randomly and equally divided into three groups (*n* = 5 per group) as follows: 1) Sham group: rats in the sham operation was treated with 1.0 ml saline by daily peritoneal injection for consecutive 14 days 2) BCCAO group: rats undergoing BCCAO was treated with 1.0 ml saline by daily peritoneal injection for consecutive 14 days 3) GB group: rats undergoing BCCAO was treated with 1.0 ml of GB at 1.0 mg/ml dissolved in mixed solution of Tween80, DMSO and saline (30: 100: 900) by daily peritoneal injection for consecutive 14 days. YZ20P6 microscopes (66 Vision Technology Co., Ltd. Suzhou, China) were used in animal model establishing experiments.

## Open Field Test

The open field test was used to reflect the autonomous behavior and exploratory behavior of experimental animals in an unfamiliar environment (Zhang et al., 2020b). The spontaneous exploratory movement activity of rats in a certain area was observed and measured. The rat was placed in a 50 cm × 50 cm×40 cm open field reaction box, the inner wall was painted black, and the bottom surface was divided into multiple small squares. According to the track of the rat's movement and behavior, the software data was automatically collected, and the experiment time was 10 min. The average movement distance and average movement speed of horizontal movement were calculated.

## The Morris Water Maze Test

The Morris water maze (MWM) test detected the spatial learning and memory ability of rats (Higaki et al., 2018). The test was divided into two parts: 1) Place navigation test: rats were placed in water pool from four different quadrant and the time period of rats climbing onto the platform in the center of quadrant from entering the water was recorded as escape latency. If the rats can not find the platform within 90 s of swimming, they were gently guided to stay the platform for 15 s before being removed from the pool. 2) Space exploration test: On the sixth day, the platform was removed, and the rats were back into the pool from the quadrant opposite to the quadrant where the platform was located, and their stay time in the original platform quadrant and the number of times they traversed the platform position were recorded in 90 s.

## Y-Maze Test

According to the previous report (Mori et al., 2017), Y-maze test consisted of three similar arms that were all placed at 120° to each other, with a triangular central zone. When the rats were placed back into the apparatus, one of the arms with lighted lamp had no electricity and the remaining two arms were electrified. Rats were shocked by electricity at 0.2 mA, 500 ms, every 1.5 s. Thus, in order to avoid being shocked, the rats had to move from the electrical arm arena to lighted arm without electricity. A camera above the maze recorded escape latency time of the rats spent in moving from (previously blocked) arm to the non-electrical arm with light on was determined. Rats completed an active avoidance when the escape latency was less than 10s, and the number of active avoidance times (correct times) was recorded.

## Nissl Staining

According to the previous report (Jiang et al., 2019), the rat brain was taken and fixed with 4% paraformaldehyde solution for 5 days, and 30% sucrose paraformaldehyde solution was used for precipitation. The fixed brain tissue was cut into slice at 20 μm and soaked it in PBS and attached into the gelatin glass slide for drying. After dehydrating with 100% alcohol for 10 min, 95% alcohol for 10 min, distilled water for 10 s, 0.1% methyl violet solution was used to stain brain. The slide was covered with a neutral resin for taking pictures under the microscope (OLYMPUS Company, Japan).

## Immunofluorescence Staining

For immunofluorescence staining, after undergoing a series of treating processes including dewaxing, hydration, antigen repairing, cell membrane perforation, inactivation of endogenous enzymes and blocking, brain sections were incubated sequentially with the primary antibody solution and fluorophore conjugated secondary antibodies (Su et al., 2019). The brain tissue slides were observed with the aid of DMI4000B fluorescence microscope from Leica company (Germany).

## Western Blot Assay

According to the previous report (Niu et al., 2020), proteins were first separated according to size or other physical properties by gel electrophoresis, and then transferred to a polyvinylidene fluoride (PVDF) membrane. After blocking with 1% BSA, the polyvinylidene fluoride membrane was incubated with primary antibodies at 4°C overnight. The membrane was washed by TBST, followed with secondary antibody for 1 h. Finally, the level of the targeted proteins was photographed and analyzed using a UVP gel analysis system (iBox Scientia 600; UVP, LLC., CA, United States).

## Results

### Ginkgobalide B Improved Survival of Oxygen-Glucose Deprivation Treated SH-SY5Y Cells by Attenuating Inflammatory Response via TLR4/NF-κB Pathway

**FIGURE 2:**
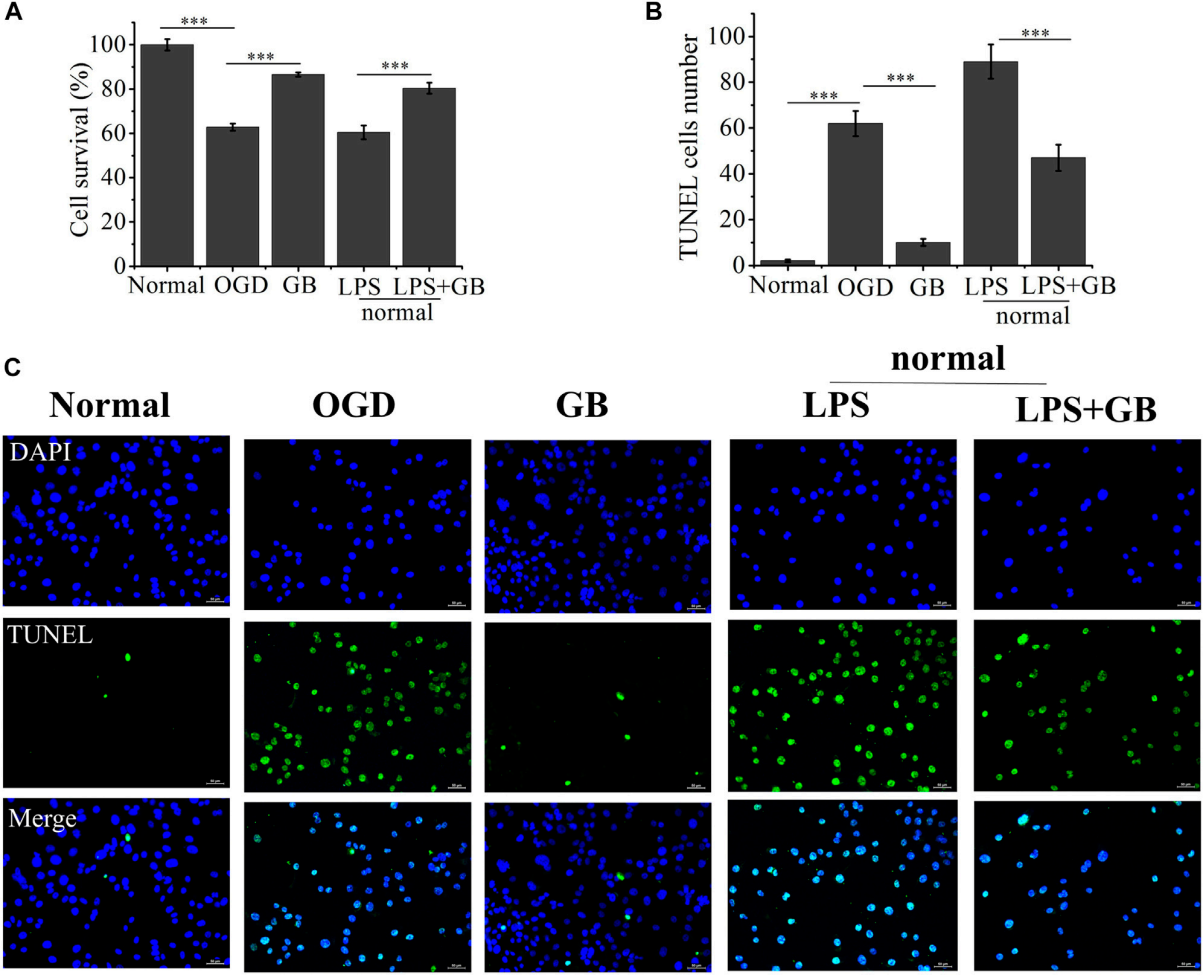
**(A)** The cell viability rate of Oxygen-Glucose Deprivation (OGD) cells treated with Ginkgobalide B (GB) and normal cells treated with Lipopolysaccharides (LPS) and the combination of LPS and GB. Data represented the mean ± SD (*n* = 5), ***p < 0.001 **(B)** Quantitative analysis on the number of TUNEL-positive of OGD cells treated with GB and normal cells treated with LPS and the combination of LPS and GB. Data represented the mean ± SD (*n* = 3), ***p < 0.001 **(C)** The images of TUNEL positive cells in different groups, the scale bar is 50 μm and applies to all figure parts.

We first investigated GB mediated survival restoration of OGD treated SH-SY5Y cells using MTT, TUNEL assay and western blot. As shown in Figure 2, MTT results demonstrated that after treated with OGD, cells were seriously damaged by decreasing their viability to 62.81%. On the contrary, the addition of GB reduced OGD induced cell death and the survival rates of cells were enhanced to 86.53%. To further verify whether GB can reduce inflammation and improve cell survival rate, LPS was added to induce cells inflammation and cells viability decreased to 60.46%. On the contrary, cell viability increased to 80.41% after adding GB. Being similar with MTT results, compared with control group, a stronger green fluorescent intensity was observed and the number of TUNEL-positive cells was significantly increased in OGD treated group, indicating that OGD aggravated the death of these cells. In the mean time, after treated with GB, less TUNEL-positive cells were visualized, suggesting that GB reduced DNA damage within OGD treated cells and effectively inhibited cell injury and death. All results confirmed that GB did exert a potential neuroprotective role and alleviated the OGD mediated cell injury.

**FIGURE 3:**
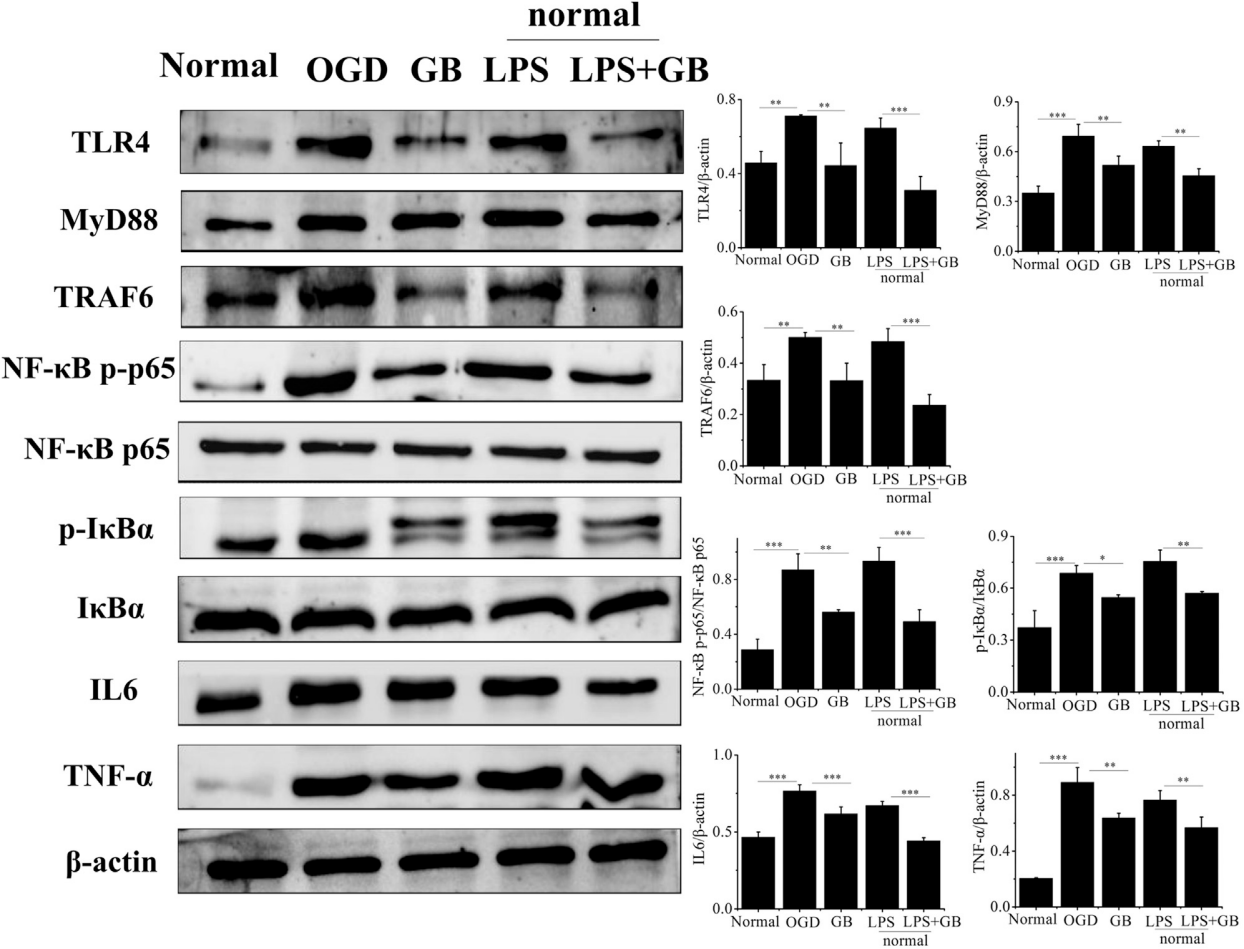
Detection of the protein levels of NF-κB p65, NF-κB p-p65, IκBα, p-IκBα, TLR4, MyD88, TRAF6, TNF-α and IL-6 by western blotting. Data represented the mean ± SD (*n* = 3), *p < 0.05, **p < 0.01, ***p < 0.001.

As shown by western blot analysis in Figure 3, we investigated whether GB reversed the cell survival by inhibiting OGD-induced inflammation of neurons. It was found that compared with normal SH-SY5Y cells, OGD activated inflammation in SH-SY5Y cells by inducing the higher expression of TLR4, and further activating adaptor molecules such as MyD88 and TRAF6 which were critical for TLR4 to activate downstream signaling pathways and induce inflammatory response (Bo et al., 2019). Furthermore, OGD activated NF-κB signaling pathways as evidenced by increasing ratio of p-IkBa/IkBa and NF-κB p-p65/NF-κB p65, and secreting a number of inflammatory factors including TNF-α and IL-6. On the contrary, compared with that from untreated OGD-stimulated SH-SY5Y cells, GB prohibited the expression of TLR4, MyD88 and TRAF6. At the same time, the phosphorylation of IkBa and NF-κB p65 appeared to be reduced, thus inducing less release of neurotoxic soluble factors and pro-inflammatory cytokines characterized by the lower expression levels of TNF-α and IL-6. We next explored the specific role of TLR4 on regulating NF-κB signaling and judged whether the neuroprotective effects of GB was related with TLR4-mediated inflammatory NF-κB signaling regulation in cells. To identify whether TLR4 mediated NF-κB signaling regulation in cells, we treated normal SH-SY5Y cells with additional lipopolysaccharides (LPS) for amplifying TLR4 expression. It was found that the addition of LPS significantly induced the apoptosis by reducing the viability of cells and increasing the number of TUNEL-positive cells. LPS largely increased the expression of TLR4 and its two adaptor molecules such as MyD88 and TRAF6 in cells, thus leading to the activation of downstream NF-κB signaling pathways by significantly up-regulating the ratio of p-IkBa/IkBa and NF-κB p-p65/NF-κB p65. It further confirmed that TLR4 mediated NF-κB signaling up-regulation induced the aggravation of neuronal injury in cells. The exposure of GB to LPS pretreated cells inhibited LPS-induced expression of TLR4, MyD88, TRAF6 and blocked LPS-induced phosphorylation of IkBα and NF-κB p65 in cells. We also found that GB reduced the expression of NF-κB targeted pro-inflammatory cytokines, such as IL-6 and TNF-α in LPS-stimulated cells. As a result, GB contributed to the reversal of LPS mediated neuronal injury in cells by enhancing the viability of cells and reducing the number of TUNEL-positive cells. Taken together, all results indicated that GB-mediated inhibition of TLR4 abolished LPS induced activation of NF-κB and reduced cell damage by reducing the TLR4/NF-κB mediated neuroinflammation in SH-SY5Y cells.

### Open Field Test and Y- Maze Test

**FIGURE 4:**
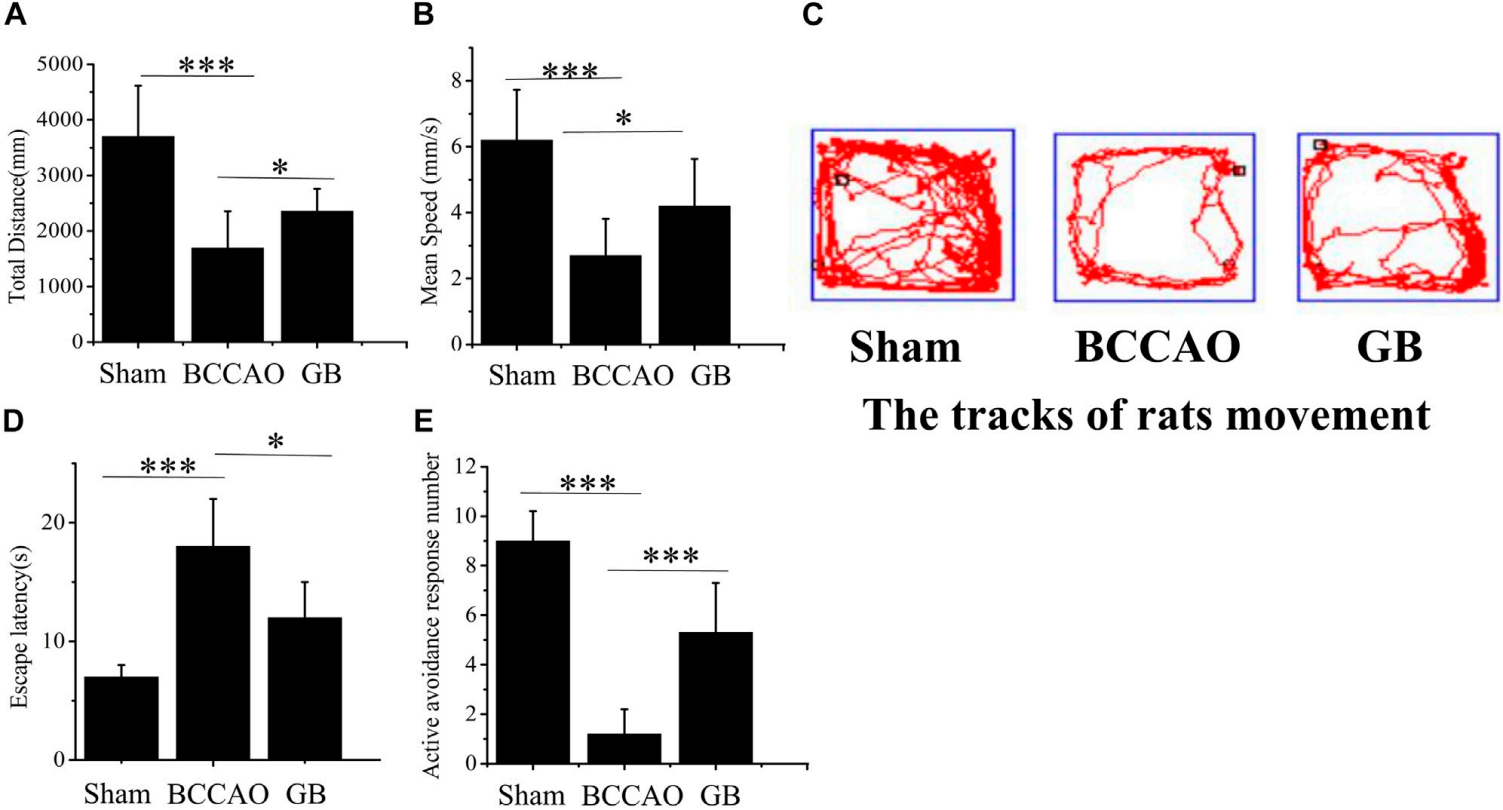
Effects of GB on movement activity and memory ability in BCCAO rats. In open field test, the change of the total distance **(A)** and mean speed **(B)** in sham group, BCCAO group and GB treated BCCAO group. Data represented the mean ± SD (*n* = 5), *p < 0.05, ***p < 0.001 **(C)** Track diagram of rats movement in the Sham, BCCAO model and GB treated BCCAO groups. Y-maze test was used to analyze working memory in rats **(D)** Escape latencies analysis. Data represented the mean ± SD (*n* = 5), *p < 0.05, ***p < 0.001 **(E)** Active avoidance response number analysis. Data represented the mean ± SD (*n* = 5), *p < 0.05, ***p < 0.001.

In the open field test, the total distance, speed of SD rats’ movement within 10 min and movement trajectory graph in different groups were further analyzed for evaluating the movement activity of rats. It showed that in Figures 4A–C, in the sham group, total distance and mean running speed were 3701.09 ± 456.55 mm and 6.17 ± 0.77 mm/s, respectively. The total distance and average speed in BCCAO group were 1641.79 ± 362.54 mm and 2.74 ± 0.61 mm/s. Compared with the sham group, movement ability of rats was significantly reduced after treatment of BCCAO. Total distance and mean running speed were significantly reduced. It indicated that the treatment of BCCAO induced the movement disorder in rats. On the contrary, the total distance and average speed in GB group were 2529.62 ± 210.37 mm and 4.21 ± 0.35 mm/s in turn. It suggested that the intraperitoneal injection of GB improved autonomous movement activity of rats with BCCAO by exhibiting longer total distance and faster mean running speed. In order to test memory ability of rats, The Y-maze test was performed by checking the escape latency and active avoidances response number in three groups after undergoing electric shock. It demonstrated that compared with the sham group, the memory function was seriously damaged in the BCCAO model group. Escape latency was prolonged and active avoidance response number was significantly reduced. In the meanwhile, GB significantly alleviated the BCCAO induced working memory impairment of BCCAO rats by reducing escape latency and increasing active avoidance response number. (Figures 4D,E).

**FIGURE 5:**
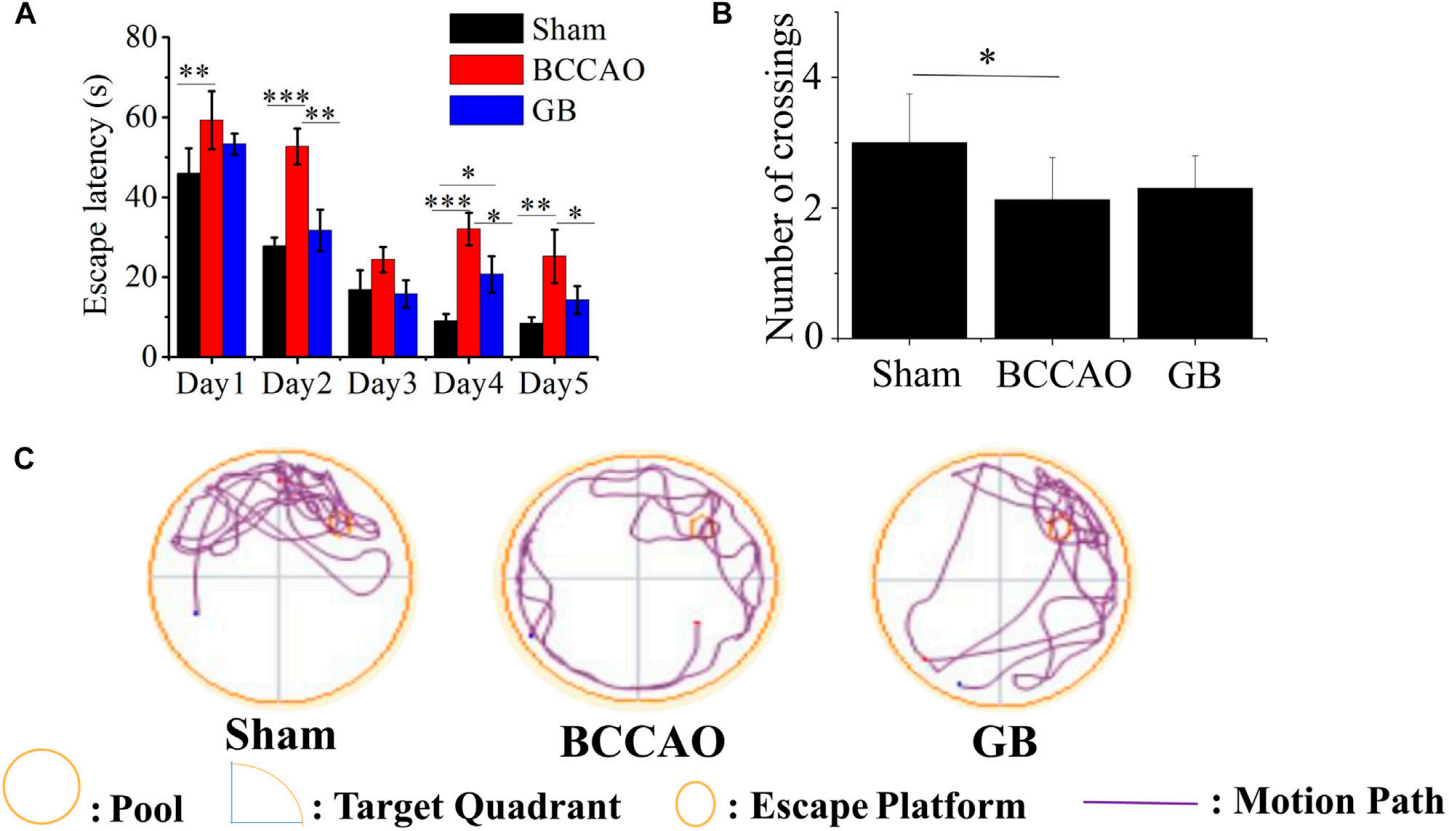
Effects of GB on improving spatial learning and memory in BCCAO rats **(A)** Escape latencies analysis. Data represented the mean ± SD (*n* = 5), *p < 0.05, **p < 0.01, ***p < 0.001 **(B)** The platform crossing number in the spatial probe test. Data represented the mean ± SD (*n* = 5), *p < 0.05 **(C)** Mapping of swimming location of rats in water maze.

Morris water maze test was also used to access spatial learning and memory of rats in three groups. The results showed that in the first 5 days, with the extension of training time, the escape latency for each group to find the platform gradually was shortened. Compared with the BCCAO model groups, the escape latency for GB treated BCCAO model group was significantly shorter (Figure 5A). After removing the platform, the number of crossings of three groups on the sixth day was analyzed. Compared with the sham group, the number of crossings in the BCCAO model group was decreased (Figure 5B). On the contrary, GB increased the number of crossings as compared to that in the BCCAO model. In view of judging the positioning trajectory diagram on the sixth day, it was found that the rats in the BCCAO group can not quickly find the quadrant where the platform was located and rotated around the wall in four quadrants. After administration of GB, the memory of rats was also significantly improved by focusing on locating the targeted quadrant and being apt to find the platform (Figure 5C). It showed that GB can relieve the spatial learning and memory decline induced by VD and had a certain positive effect on improving cognitive function.

**FIGURE 6:**
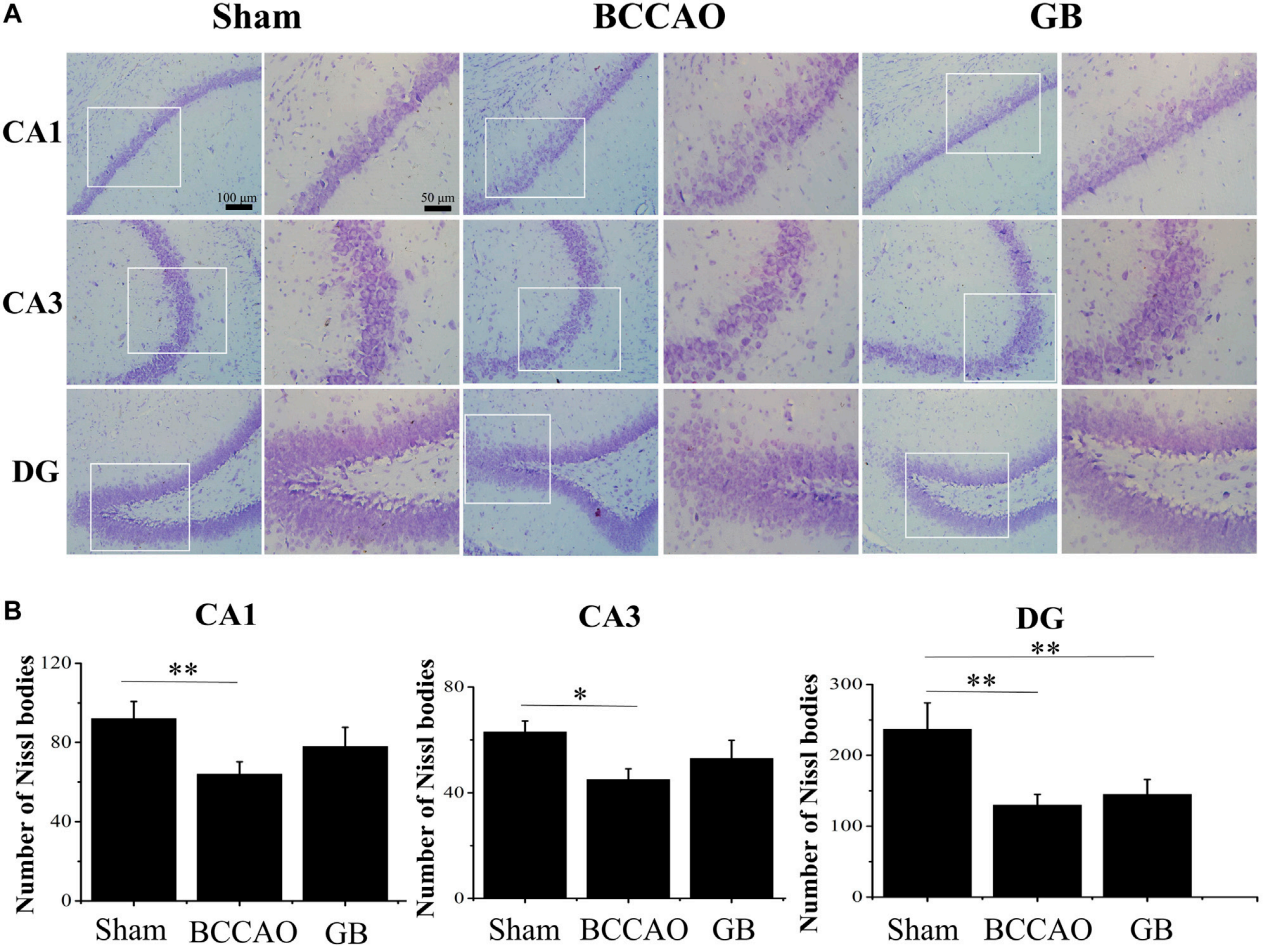
Nissl staining of CA1, CA3, DG region of rat hippocampus of sham group, BCCAO group and GB treated BCCAO group. The Nissl body was stained purple-blue. **(A)** Representative Nissl staining images in hippocampus of sham group, BCCAO group and GB treated BCCAO group. **(B)** Quantitative analysis of the number of Nissl bodies in hippocampus of sham group, BCCAO group and GB treated BCCAO group. Data are expressed as means ± SD (n = 3), *p < 0.05, **p < 0.01. Scale bar: 100 and 50 μm.

It is well known that neurons in the hippocampus play an important role on regulating the main movement, learning and memory functions. Therefore we used Nissl staining and immunofluorescence staining for evaluating BCCAO induced neuron damage. As shown in Figure 6, the neurons in the CA1, CA3 and DG regions of the hippocampus of rats in the sham group were cone-shaped and closely arranged. In the meanwhile, more Nissl bodies were observed and the cells were closely arranged, regular in shape, and darker in color in the cytoplasm. On the contrary, the Nissl bodies in the hippocampus of rats in the BCCAO model group were loose, sparse, irregular in shape, and the number of Nissl bodies was significantly decreased, indicating that BCCAO induced the neuronal damage in the CA1, CA3 and DG regions of the hippocampus of rats as compared to sham group. In GB treated BCCAO model group, the pyramidal cells restored to be neatly distributed and maintained integrity of basic neuronal structure. Moreover, it induced the higher number of Nissl bodies in the CA1, CA3 and DG regions of the hippocampus of rats. Taken together, GB showed significantly greater protective effects and reversed BCCAO induced neuronal damage in hippocampus.

**FIGURE 7:**
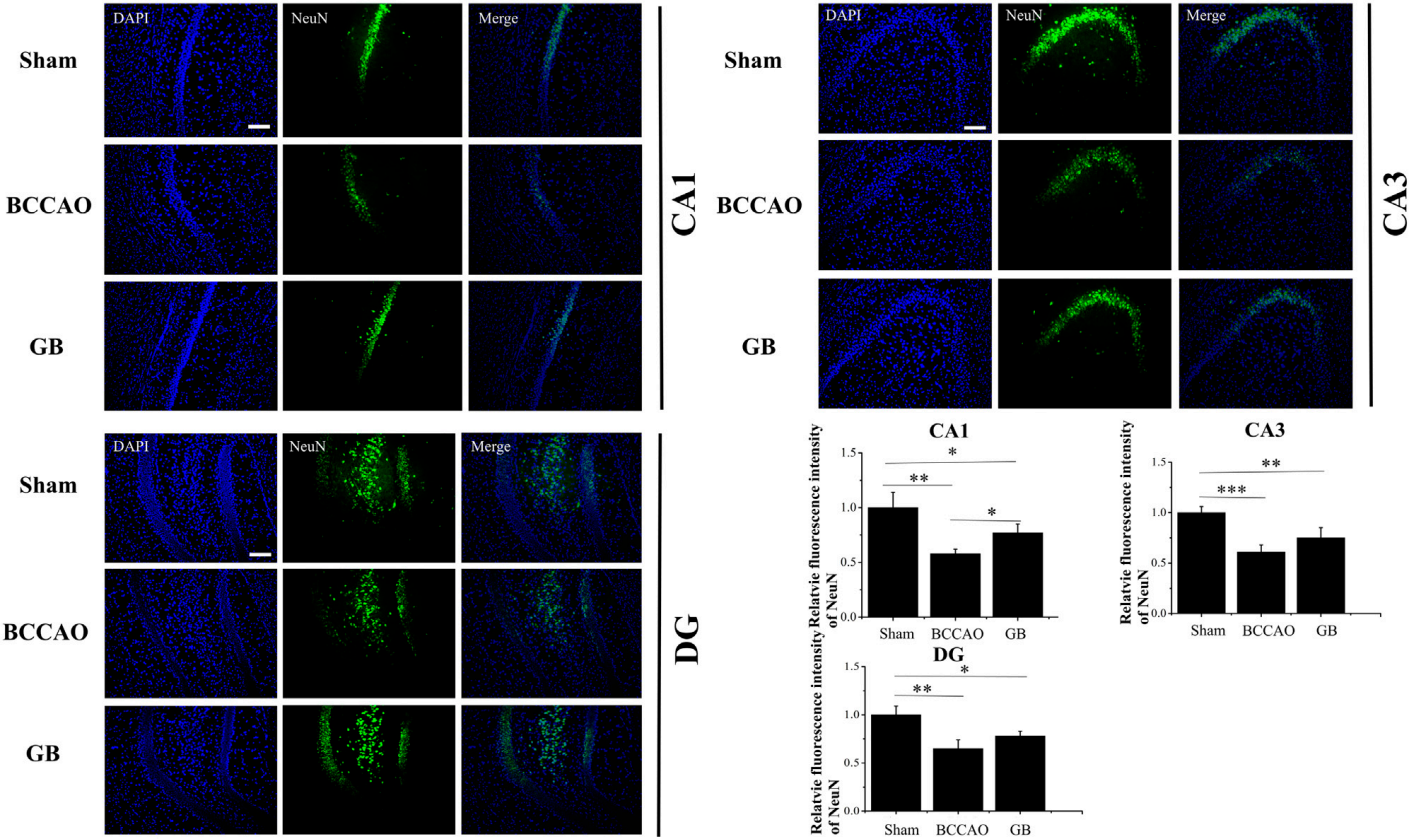
Representative immunofluorescence staining for NeuN positive cells in hippocampus of sham group, BCCAO group and GB treated BCCAO group. NeuN antibodies were used to stain neurons in the hippocampus. DAPI (blue) was used as a nuclear marker. Data are expressed as means ± SD (*n* = 3), *p < 0.05, **p < 0.01, ***p < 0.001. The scale bar is 200 μm and applies to figure 7.

We used immunofluorescence staining to observe neurons and the number of neurons in hippocampus was counted by comparing the fluorescence intensity of NeuN. We observed in Figure 7 that in the BCCAO model, the fluorescence intensity of hippocampal neurons in the CA1, CA3 and DG areas was weaker, and the neuron loss was more obvious, indicating that BCCAO reduced the number of NeuN-positive neurons and VD can cause neurons death. Importantly, GB can reduce the apoptosis of neurons in hippocampus by showing the highest number of NeuN-positive neurons in the CA1, CA3 and DG. All results demonstrated that GB indeed further contributed to neuroprotection and suppressed the apoptosis of neurons in hippocampus.

### Western Blot Analysis

**FIGURE 8:**
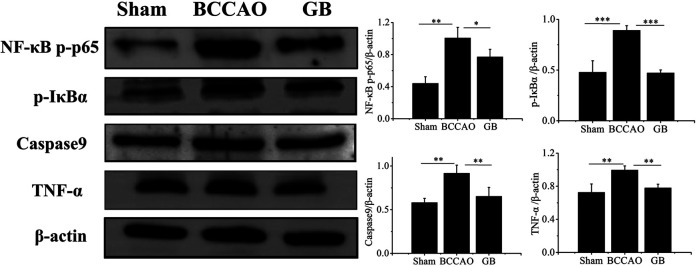
Detection of the protein levels of NFκB p-p65, p-IκBα, Caspase9 and TNF-α in brain tissue by western blotting. Data represented the mean ± SD (*n* = 3), *p < 0.05,**p < 0.01, ***p < 0.001.

In order to determine whether GB treatment could inhibit TLR4/NF-κB mediated inflammation response in BCCAO rats, we detected the expression level of NF-κB p-p65 and p-IκBα, pro-inflammatory cytokines TNF-α following pre-treatment and post-treatment. As Figure 8 indicated, BCCAO activated NF-κB mediated neuroinflammation by increasing NF-κB p-p65 and p-IκBα and secreting more TNF-α. Importantly, GB significantly reduced the level of NF-κB p-p65, p-IκBα and TNF-α. These founding suggested that GB can reduce the expression of TNF-α and NF-κB related proteins, slow down the inflammatory cascade reaction and suppress the cell apoptosis for treating VD, which be related to the inhibition of NF - κB signaling pathway. GB effectively reduced the NF-κB mediated neuroinflammation in BCCAO rats. Furthermore, GB regulated apoptosis-related proteins such as caspase9, thus exerting its neuroprotective effect.

## Discussion

Vascular dementia (VD) leads to vascular cognitive impairment (VCI) with the syndrome from mild cognitive impairment to dementia caused by cerebrovascular disease risk factors. At present, the clinical treatment of Alzheimer’s disease and memory deficits includes acetylcholines such as Ach prodrugs, cholinesterase inhibitor-tacrine, piracetam for enhancing brain metabolism, Glutamate receptor modulator-Mematitine, Nicergoine for inhibiting platelet aggregation and calcium antagonist-Nimodipine (Pantoni., 2004; Farooq, et al., 2017). Enhanced inflammatory response is often occurred in VD and many regulators such TNF-α and NF-κB as important mediators of inflammation are involved in the pathogenesis of VD. GB is a natural PAF-R antagonist, which can reduce inflammation, increase cerebral blood flow, improve cerebral vascular circulation, prevent thrombosis caused by platelet aggregation and promote learning function of memory (Gu et al., 2012; Chen et al., 2020; Xiang et al., 2020). Studies have shown that GB has a neuroprotective effect on neuronal cells deprived of oxygen and glucose (Yang et al., 2018) and exerts an antioxidant effect on oxidative stress caused by transient focal cerebral ischemia (Liu et al., 2019). It regulates the polarization of microglia for enhancing protective effect on ischemic stroke (Shu et al., 2016) and inhibits excitotoxicity by regulating the imbalance between excitatory amino acids and inhibitory amino acids, thereby attenuating cerebral ischemic injury (Yang et al., 2011). GB also can significantly inactivate pro-inflammatory factors through the NF-κB pathway, thereby reducing cell damage and apoptosis (Zhang et al., 2018; Li et al., 2020). In this study, we investigated the therapeutic effect of GB on alleviating learning and memory impairment in rats with VD. The possible neuroprotective mechanism of GB on regulating TLR4/NF-κB signaling pathway was further explored.

To simulate the pathological process of chronic cerebral ischemia in patients with VD, we adopted the permanent bilateral common carotid artery occlusion model to perform our experiment. Through the chronic cerebral hypoperfusion, the vulnerable areas of brain tissue such as hippocampal atrophy and cortex appeared gradual ischemia and hypoxia damage, resulting in VD like the decline of learning and memory ability and serious cognitive dysfunction.

In order to investigate whether GB can improve the learning and memory function of VD rats, a series of testing models such as open field test, Morris water maze and Y- maze test were performed to detect the behavioral changes of VD rats induced by GB. We found that GB improved the cognitive level of rats with VD by protecting hippocampal neurons and reducing apoptosis. Compared with BCCAO model, after treated with GB, total distance and mean running speed were significantly enhanced in open field test. GB also reduced escape latency and increased active avoidance response number in Y- maze test. Furthermore, the escape latency was significantly reduced and the number of crossings was increased in GB treated BCCAO model group in Morris water maze. Nissl staining and immunofluorescence results demonstrated that compared with BCCAO model, the number of neurons was significantly increased in GB treated BCCAO group, the hippocampal structure was clearly viewed, dense neurons with plenty cytoplasm and deep-dyed big nuclei were observed.

Since GB can improve the learning and memory function of VD rats, we further make clear the relation between its protective effect of GB and regulation of TLR4/NF-κB signaling pathway. In OGD group, TLR4 was predominantly activated and its adaptor proteins such as MyD88 and TRAF6 were also activated. Finally transcription factors NF-κB were up-regulated and these transcription factors induced expression of proinflammatory cytokine gene. Therefore, it was concluded that BCCAO induced OGD may upregulated the TLR-4-dependent NF-κB signaling pathway to induce neuron injury. On the contrary, the expressions of TLR-4, MyD88, TRAF6, NF-κB p-p65, p-IκBα were reduced and the levels of TNF-α and IL-6 were decreased in GB treated OGD group. In addition, GB significantly reduced the level of NF-κB p-p65, p-IκBα and TNF-α *in vivo*. It indicated that NF-κB signaling pathway was involved in the development of VD and GB ameliorated VD induced neuronal injury through inhibiting TLR4/NF-κB signaling pathway.

## Conclusion

We used bilateral common carotid artery occlusion method to establish VD rat model. The protective effect of GB on improving the learning and memory function of VD rats was investigated. Furthermore, the possible mechanism of GB mediated neuroprotection on regulating NF-κB pathway in the development of VD was further explored. All data confirmed that GB significantly reduced BCCAO induced neuroinflammation and improved neuron survival by reducing the secretion of inflammatory factors via downregulating TLR4/NF-κB pathway, thus alleviating learning and memory impairment in rats with VD.

## Data Availability

The raw data supporting the conclusion of this article will be made available by the authors, without undue reservation.

## Abbreviations

GB, Ginkgobalide B; BCCAO, bilateral common carotid artery occlusion; VD, vascular dementia; OGD, oxygen-glucose deprivation; MWM, Morris water maze.
